# Treatment for acute uncomplicated diverticulitis without antibiotherapy: systematic review and meta-analysis of randomized clinical trials

**DOI:** 10.1097/JS9.0000000000000307

**Published:** 2023-04-10

**Authors:** Alba Correa Bonito, Carlos Cerdán Santacruz, Marcello Di Martino, Lara Blanco Terés, Álvaro Gancedo Quintana, Elena Martín-Pérez, Sebastiano Biondo, Javier García Septiem

**Affiliations:** a Colorectal Surgery Unit, General and Digestive Surgery Service; b General and Digestive Surgery Service, Hospital Universitario de La Princesa, Madrid; c General and Digestive Surgery – Colorectal Unit, Bellvitge University Hospital, Barcelona, Spain; d Ospedale Cardarelli, A.O.R.N, HBP Department, Napoli, Campania, Italy

**Keywords:** acute uncomplicated diverticulitis, antibiotherapy, conservative management

## Abstract

Use of antibiotics in selected cases of acute uncomplicated diverticulitis (AUD) has recently been questioned.

**Objective:**

The aim of this study is to examine the safety and efficacy of treatment regimens without antibiotics compared with that of traditional treatments with antibiotics in selected patients with AUD.

**Data sources:**

PubMed, Medline, Embase, Web of Science, and the Cochrane Library

**Methods:**

A systematic review was performed according to PRISMA and AMSTAR guidelines by searching through Medline, Embase, Web of Science, and the Cochrane Library for randomized clinical trials (RCTs) published before December 2022. The outcomes assessed were the rates of readmission, change in strategy, emergency surgery, worsening, and persistent diverticulitis.

**Study selection:**

RCTs on treating AUD without antibiotics published in English before December 2022 were included.

**Intervention:**

Treatments without antibiotics were compared with treatments with antibiotics.

**Main outcome measures:**

The outcomes assessed were the rates of readmission, change in strategy, emergency surgery, worsening, and persistent diverticulitis.

**Results:**

The search yielded 1163 studies. Four RCTs with 1809 patients were included in the review. Among these patients, 50.1% were treated conservatively without antibiotics. The meta-analysis showed no significant differences between nonantibiotic and antibiotic treatment groups with respect to rates of readmission [odds ratio (OR)=1.39; 95% CI: 0.93–2.06; *P*=0.11; *I* ^2^=0%], change in strategy (OR=1.03; 95% CI: 0.52–2,02; *P*=0.94; *I* ^2^=44%), emergency surgery (OR=0.43; 95% CI: 0.12–1.53; *P*=0.19; *I* ^2^=0%), worsening (OR=0.91; 95% CI: 0.48–1.73; *P*=0.78; *I* ^2^=0%), and persistent diverticulitis (OR=1.54; 95% CI: 0.63–3.26; *P*=0.26; *I* ^2^=0%).

**Limitations:**

Heterogeneity and a limited number of RCTs.

**Conclusions:**

Treatment for AUD without antibiotic therapy is safe and effective in selected patients. Further RTCs should confirm the present findings.

## Introduction

Highlights

- Acute uncomplicated diverticulitis (AUD) is no longer considered an infectious disease.
- In selected cases, AUD can be treated without antibiotherapy.
- Treatment without antibiotherapy in selected patients is safe and feasible.

Symptomatic diverticular disease is an extremely common disease in occidental and industrialized countries in recent times. Its prevalence has increased in the last few decades due to an increased number of older individuals in the population. Approximately 50% of the population aged over 60 years have colonic diverticula^1^. Although in most cases they are asymptomatic, 4–25% of them present an episode of acute diverticulitis at some point in their lives^2^,^3^.

Among symptomatic patients, 75% present AUD and are prescribed conservative treatments^4^,^5^. Until a few years ago, general recommendations for such patients were hospital admissions, nil per os, and administration of oral or parenteral antibiotics.

Although initially considered as an intra-abdominal infection secondary to a microperforation at any diverticula, for which antibiotic treatment was mandatory, recently, AUD has been considered as merely an inflammatory process^6^. Based on this new physiopathological conception of AUD, Hjern *et al*.^7^ first reported the safety of a conservative treatment of AUD that did not involve antibiotic therapy. Recently, the possibility of treating these patients without antibiotics has experienced a growing interest and some research has been done, including that in outpatient regimes and some randomized clinical trials (RCTs)^8^–^13^. The conclusions of most studies have been similar, agreeing on the safety of avoiding antibiotics in certain cases of AUD. Consequently, the latest clinical guidelines on this topic reflect the recommendation for antibiotic-free treatment in selected cases of AUD^14^–^16^.

However, despite these recommendations, the implementation of such treatments in clinical practice is less than desirable. The unnecessary administration of antibiotics could result in adverse effects for the patients, risk of resistance, and increased costs.

This systematic review and meta-analysis aims to compare the safety and effectiveness of conservative treatment without antibiotherapy to that of antibiotic treatment for patients with AUD.

## Materials and methods

### Search strategy

The search was undertaken according to the Preferred Reporting Items for Systematic Reviews and Meta-Analyses (PRISMA & AMSTAR) guidelines^17^,^18^. Two researchers (A.C.B. and C.C.S.) systematically searched Medline, Embase, Web of Science, and the Cochrane Library for reports published before December 2022, not limited to those in English. Specific research equations were formulated for each database using a combination of the following predefined keywords and/or MeSH terms: ‘diverticulitis,’ ‘diverticular disease,’ ‘antibiotics,’ and ‘antimicrobials.’

### Study selection

**Figure 1:**
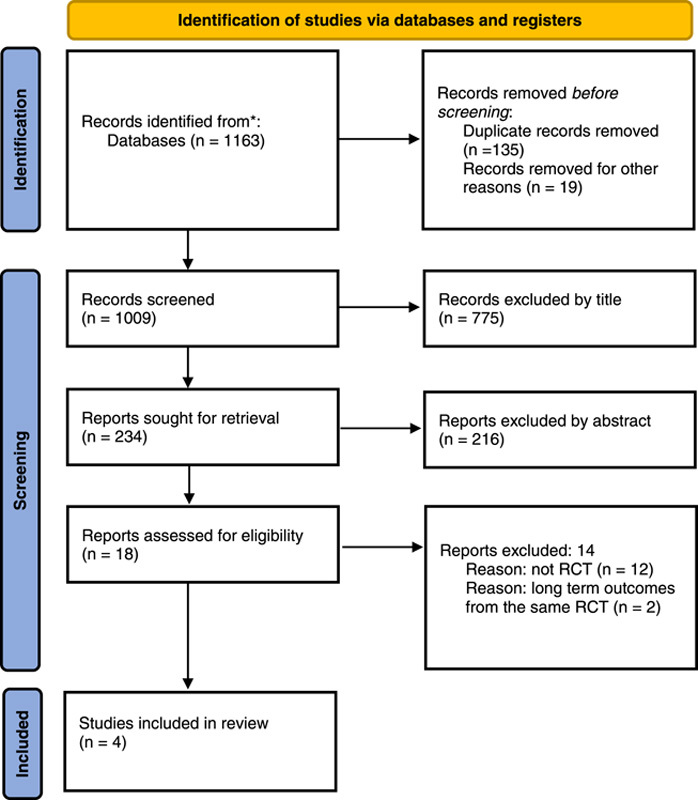
PRISMA flow diagram. PRISMA, Preferred Reporting Items for Systematic Reviews and Meta-Analyses; RCT, randomized clinical trial.

Studies included in the review were those that met the following criteria: (a) studies on AUD patients aged 18 years or over; and (b) randomized controlled designs comparing treatments of acute diverticulitis with and without antibiotics. Experimental studies on animal models, observational studies, reviews, editorials, and comments were excluded. When duplicate reports from the same study were identified, those which analyzed long-term outcomes were excluded^8^,^9^. The full text of each article included in the initial review was assessed. The selection process is detailed in the PRISMA^17^ flow diagram (Fig. 1).

### Data collection

Two researchers (A.C.B. and C.C.S.) independently assessed the abstracts of the selected studies to determine their eligibility. Full articles were subsequently selected for further assessment. The therapeutic options included treatments with or without antibiotics. The extracted data included the country of study, year of publication, design, number of participants, age, radiological severity score, percentage of patients with antibiotic treatment, percentage of patients without antibiotic treatment, duration of follow-up in months, rates of mortality, complications during follow-up, rates of readmission and recurrence, rates of surgical intervention during follow-up, rates of change of strategy and length of hospital stay.

Disagreements over data extraction were resolved by consensus between the two authors and by a discussion with two other authors (M.D.M. and J.G.S.) in the absence of consensus.

### Outcome variable definition

Primary outcomes:

- Readmission was defined depending on the initial regime offered to the patients. For the patients admitted to the hospital, a new admission to the hospital after discharge was considered as readmission. For the patients treated with the outpatient regime, any new visits to the emergency department were considered as readmission.
- Change in strategy was defined as any deviation from the initially designed management: initiation of antimicrobial treatment in the case of previous management without antibiotherapy; the necessity of surgery or percutaneous drainage or the necessity of hospitalization in the case of outpatient management.

Secondary outcomes:

- Emergency surgery was defined for patients that needed emergency surgery or percutaneous drainage.
- Worsening was defined in terms of the number of patients whose episode progressed from a noncomplicated episode to an abscess, fistula, or perforation.
- Persistent diverticulitis was defined as the rate of patients that had persistent symptoms during the first month of follow-up.

### Evaluation of studies and statistical analysis

Two researchers (A.C.B. and C.C.S.) independently evaluated the included studies for quality assessment according to the Cochrane Handbook for Systematic Reviews of Interventions^17^. The data were analyzed using the RevMan statistical software Version 5.4 and presented as medians and proportions along with a corresponding minimum–maximum range. To estimate proportions, we used the odds ratios (ORs) with their respective 95% CIs with a random effects model to take into account the heterogeneity of the estimates. Differences in dichotomous variables were calculated using standardized mean differences with 95% CIs. Values were considered statistically significant when *P* was less than 0.05. The overlapping of CIs was used to visually assess the heterogeneity. Heterogeneity was statistically explored with the chi-square test with significance set to a *P*-value of 0.10. The quantity of heterogeneity was measured with the *I* ^2^ statistic.

## Results

The literature search yielded 1163 studies. After removing duplicate records and screening titles and abstracts, 18 articles that satisfied the selection criteria were selected. An additional search through the references of the included studies did not yield any studies suitable for further inclusion. A total of 12 papers were excluded due to those studies not being RCTs. Among the six remaining RCTs, two^8^,^9^ were excluded because their studied populations were the same as those of two other included studies^10^,^11^, while they had different outcomes that were not related to the present study (e.g. long-term quality of life). Four papers^10^–^13^ were ultimately included in the analysis (Fig. 1).

### Study characteristics

Four trials with a total of 1809 patients were included in this study. A total of 907 patients were treated without antibiotics, whereas 902 patients were treated with antibiotics. The number of patients included from each study ranged from 180 to 623. The mean or median age ranged from 57 to 58 years, the proportion of females from 49 to 64%, and the follow-up period from 1 to 12 months. All trials involved a short-term or medium-term follow-up. Patient characteristics and inclusion and exclusion criteria for each study are shown in Tables 1 and 2.

**Table 1:** Articles included in the meta-analysis comparing outcomes.

References	Country	Type	Number of centers	N	Female (%)	Mean age	Number without antibiotics	Number with antibiotics	Follow-up (months)
Chabok *et al*.^10^	Sweden and Iceland	RCT	Multicenter	623	64	57	309	314	12
Daniels *et al*.^11^	The Netherlands	RCT	Multicenter	528	49	57	262	266	6
Jaung *et al*.^12^	New Zealand	RCT	Multicenter	178	58	58	94	84	1
Mora López *et al*.^13^	Spain	RCT	Multicenter	480	53	58	242	238	3

RCT, randomized clinical trial.

**Table 2:** Intervention, inclusion and exclusion criteria, and outcomes of each RCT.

References	Intervention	Outpatient treatment	Inclusion criteria	Exclusion criteria	Primary outcome	Secondary outcomes
Chabok *et al*.^10^	Intravenous fluid only vs. intravenous broad-spectrum antibiotics for 7 days	No	>18 years Acute lower abdominal pain with tenderness Body temperature ≥38°C at admission or during the last 12 h before admission Raised WBC and C-reactive protein level Diverticulitis on CT Informed consent	Complicated diverticulitis on CT with abscess, fistula, or free air Active immunosuppressive therapy Pregnancy Ongoing antibiotic therapy High fever, affected general condition, peritonitis, or sepsis	Complications Emergency surgery	Recurrence Length of hospital stay Abdominal pain Bowel habit
Daniels *et al*.^11^	Amoxicillin-clavulanic acid or ciprofloxacin+metronidazole if allergy for 10 days (2 days of intravenous inpatient treatment)	No antibiotic group	Left-sided uncomplicated AD according to the modified Hinchey Ambrosetti classification (Ia–Ib) Informed consent given by the patient	Previous episode of diverticulitis Proven or suspicion of colonic cancer Inflammatory bowel disease Modified Hinchey stages 2–4 Other disease with expected survival *<*6 months Contraindication to use of the study medication Pregnant, breastfeeding ASA*>*III Immunocompromised Clinical suspicion of bacteremia Inability to read/understand and fill in questionnaires Antibiotic use in the 4 weeks before inclusion	Time to recovery	Days spent outside hospital in the 6-month period Readmission rate Occurrence of complicated Diverticulitis Ongoing diverticulitis AD recurrence, Need for sigmoid resection or other (non) surgical intervention within 6 and 12 months of follow-up Serious adverse events Side-effects of initial antibiotic treatment All-cause mortality
Jaung *et al*.^12^	Intravenous cefuroxime 750 mg and oral metronidazole 400 mg and oral amoxicillin/clavulanic acid 625 mg, or placebo	No	Noncomplicate AD Informed consent given by the patient	If they met 2 of these criteria upon presentation to the hospital: temperature <36°C or >38°C, HR >90 bpm, RR >20 rpm or PaCO_2_ <32 mmHg, WBC count <4 or >12 10^9^/l Unable to give consent or answer symptom-related questions Previous drug reactions to the antibiotics used in the study Lactose allergy Used steroids for >5 days before presentation Immunomodulators or biologics NSAIDs for greater than a week before presentation >1 dose of intravenous or >2 doses of oral antibiotics during this illness but before enrollment in the study Pregnant ASA>4 CT evidence of complicated AD	Length of hospital admission (h)	Participant dropout or withdrawal rate Occurrence of adverse events Readmission within 1 week and 30 days Procedural intervention Change in serum markers of inflammation Patient-reported pain score at 12 and 24 h
Mora-López *et al*.^13^	ATB group: 875/125 mg/8 h amoxicillin/clavulanic acid and symptomatic treatment Non-ATB group: antiinflammatory and symptomatic treatment Clinical controls were performed at 2, 7, 30, and 90 days	If good symptomatic control	18–80 years Modified Neff 0 AD on abdominal CT scan No AD episode in the last 3 mo No antibiotic treatment in the last 2 weeks No significant comorbidities Immunocompetence Patient’s written informed consent Adequate cognitive capacity Adequate family support Good symptom control at the ED Maximum 1 of the following: temperature >38°C, temperature <36°C, L >12 000/ml, L <4000/ml, HR >90 bpm, RR >20 rpm, CPR >15 mg/dl	Pregnancy or breastfeeding, <18 years or >80 years, Allergy to any of the study drugs Modified Neff ≥ I AD AD episode in the last 3 months Inflammatory bowel disease Antibiotic treatment for any reason in the last 2 weeks Presence of significant comorbidities Immunodepression Absence of patient’s written informed consent Inadequate cognitive capacity Inadequate family support Poor symptom control at the ED (VAS >5) and/or systemic inflammatory response síndrome	Hospital admission	Number of ED revisits, Pain control Emergency surgery

AD, acute diverticulitis; ASA, American Society of Anaesthesiologists; ATB, antibiotic; CPR, cardiopulmonary resuscitation; CT, computed tomography; ED, emergency department; HR, heart rate; PaCO_2_, partial pressure of carbon dioxide; RR, respiratory rate.; RCT, randomized clinical trial; VAS, Visual Analog Scale; WBC, white blood cell.

### Quality assessment

**Figure 2:**
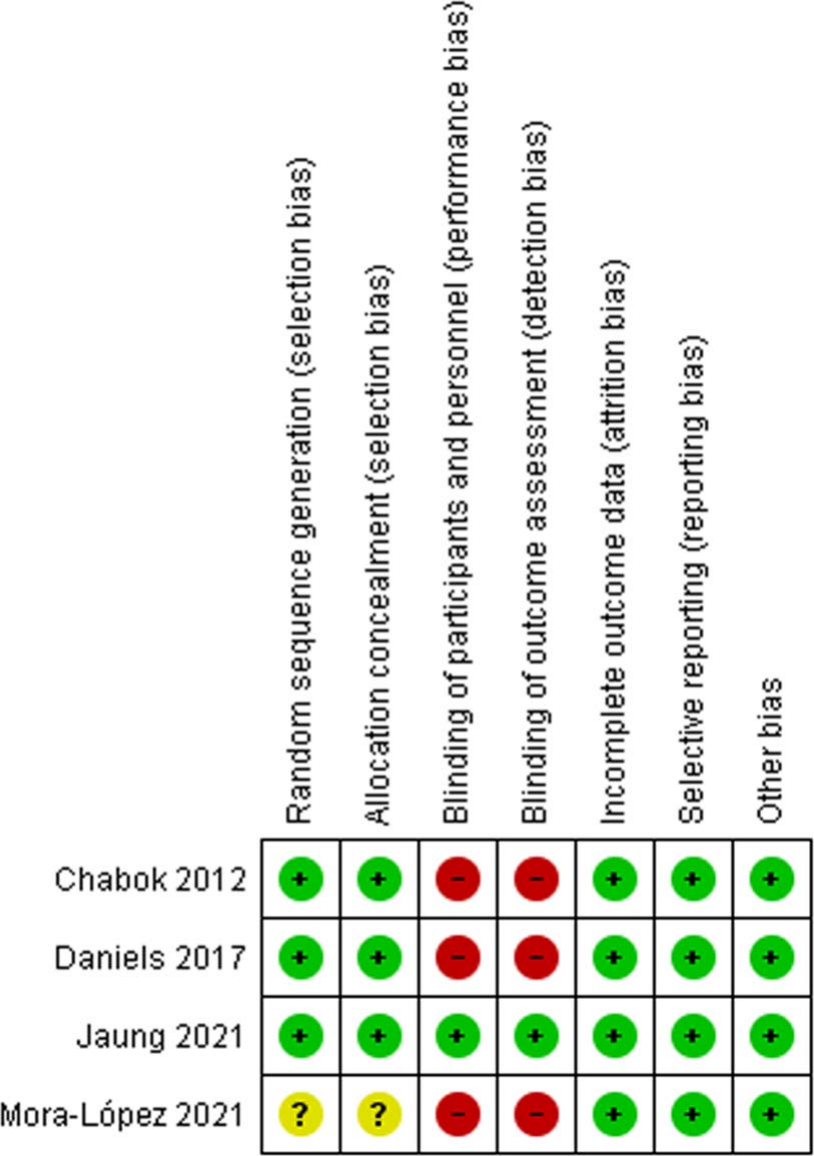
Cochrane risk-of-bias 2 assessment of randomised controlled trials included in the meta-analysi.

The results of the quality assessments of the four included studies are summarized in Figure 2. The Jaung and colleagues’ trial was the only one at low risk of bias in all domains, such as sequence generation, allocation concealment, blinding, incomplete outcome data, and selective outcome reporting. It was the only trial with a double-blinded design. The other three studies (10, 11, and 13) were at a high and unclear risk of bias from lack of blinding and lack of information on randomization and allocation methods.

### Primary outcomes

#### Readmission

**Figure 3:**
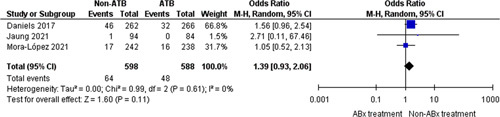
Readmission.

In the analysis of readmission rates (Fig. 3), a total of 112 events (9% of patients) were recorded from three trials^11^–^13^ (1186 participants). No difference was found between the readmission rates in the nonantibiotic group (64/589; 10.7%) and the control group (48/588; 8.1%) (OR=1.39, 95% CI: 0.93–2.06, *P*=0.11).

### Change in strategy

**Figure 4:**
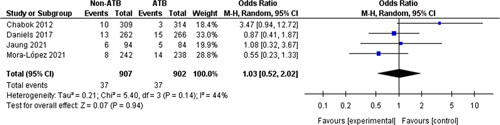
Change in strategy.

Four trials^10^–^13^ (1809 participants) reported on the change in strategy (Fig. 4) yielding a total of 112 events (4.1% of participants). A comparison of the change in strategy between the nonantibiotic group (37/907 patients; 4.1%) and the control group (37/902 patients; 4.1%) did not show any differences between the two groups (OR=1.03, 95% CI: 0.5–2.02, *P*=0.94).

### Secondary outcomes

#### Emergency surgery

**Figure 5:**
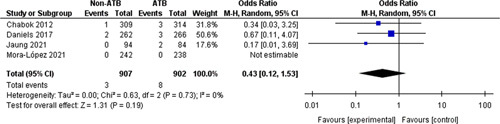
Emergency surgery.

Rates of emergency surgery were analyzed in four trials^10^–^13^ (1809 participants) with 11 events in total (0.6% of participants). In the nonantibiotic group, three of 907 patients (0.03%) needed emergency surgery, whereas, in the antibiotics group, eight of 902 patients (0.08%) needed emergency surgery. This difference was neither statistically nor clinically significant (OR=0.43, 95% CI: 0.12–1.53, *P*=0.19) (Fig. 5).

### Worsening

**Figure 6:**
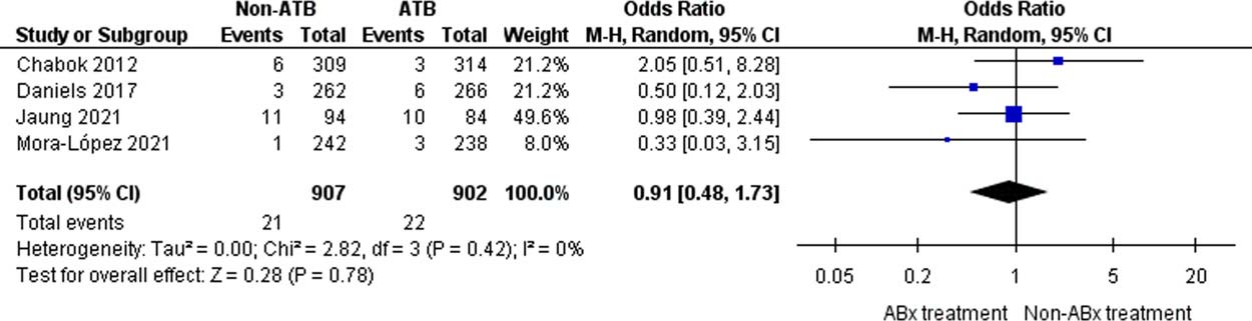
Worsening.

The analysis of the rates of clinical or radiological worsening included cases from four trials^10^–^13^ (1809 participants) with 43 events in total (2.3%). The cases of worsening comprised 21 of 907 (2.3%) patients in the nonantibiotic group and 22 of 902 (2.4%) patients in the control group (OR=0.91, 95% CI: 0.48–1.73, *P*=0.78) (Fig. 6).

### Persistent diverticulitis

**Figure 7:**
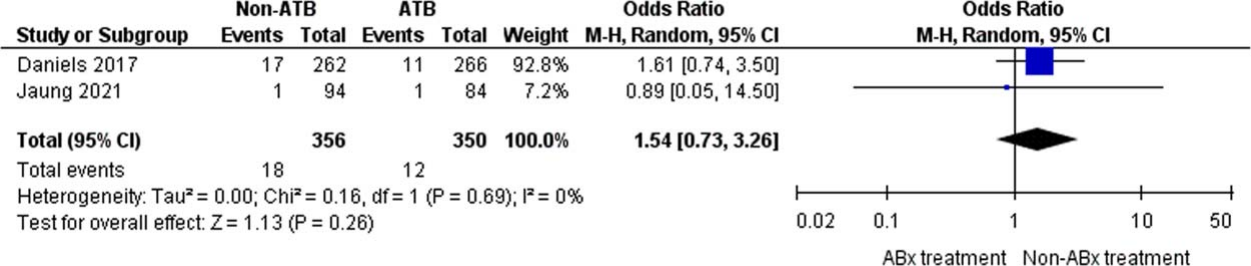
Persistent diverticulitis.

Two trials^11^,^12^ (706 participants) reported cases of persistent diverticulitis with 30 events in total (4.2% of participants presented persistent diverticulitis). A comparison of the number of patients with persistent diverticulitis in the nonantibiotic group (18/356 patients: 5.0%) with those in the control group (22/902 patients: 3.4%) did not show any differences between the two groups (OR=1.54, 95% CI: 0.73–3.26, *P*=0.26) (Fig. 7).

## Discussion

This meta-analysis conducted with 1809 patients is the first to exclusively include studies with the highest level of evidence (Level 1A of evidence according to the Oxford Centre for Evidence-Based Medicine^19^), including four RCTs^10^–^13^ that describe the short-term outcomes of AUD patients treated conservatively with and without antibiotics. The comparison between nonantibiotic treatments and traditional treatments did not show any clinically relevant or statistically significant differences in short-term outcomes. The results obtained with our methodology are consistent with those of previous systematic reviews^20^–^22^, despite the latter being based on studies with an inferior level of evidence^23^,^24^. These results show the safety and effectiveness of the therapeutic strategy approved and supported by diverse scientific societies^14^,^15^.

It is remarkable that only two RCT^12^,^13^ have been published after the inclusion of such recommendations at guidelines. The other two^10^,^11^, were published before thanks to the broadcasting of several observational studies that defended the safety of the treatment without antibiotherapy of AUD^7^.

The methodological differences among the studies are quite remarkable. There exist significant differences in the primary outcome of the studies, in-hospital versus outpatient management as well as prominently different antibiotic schemes among different trials. Such heterogeneity results in various distinguishable characteristics of the analysis, such as the huge differences among sample size calculations (180^12^ vs. 623^10^).

The variability of the antibiotics used for AUD is notable. Broad-spectrum antibiotics such as meropenem or piperacillin/tazobactam are used in cases of AUD despite clinical guidelines tending to avoid recommendations of such antibiotics for patients without risk factors and mild intra-abdominal infections^25^. Nevertheless, nonantibiotic therapy seemed to be not inferior to broad-spectrum antibiotic regimes, and no detrimental effect was found in all the variables analyzed in those studies, what is the really relevant issue that is being studied in this systematic review and meta-analysis. Such aggressive policies may entail an increased risk of the development of multiresistance in the implicated flora that can be precisely targeted by the avoidance of the systematic use of antibiotherapy.

It is important to remark that not all the studies use the same nonantibiotic therapy and, in some cases^13^, NSAIDs is the alternative to antibiotics, while in others, the regime is based in placebo^12^ or fluids intravenously^10^. This heterogeneity should be evaluated in further research to answer the question of what is the best alternative to antibiotics.

Few differences have been observed in relation to inclusion and exclusion criteria and the most relevant ones are related to the first RCT published^10^. The latest RCTs are more homogeneous at this point, with subtle differences in terms of radiological classification and clinical response to the inflammatory syndrome, but all of them include low-risk patients with AUD and without signs of clinical severity, nor radiological signs of complicated diverticulitis.

Despite the most recent evidence-based studies^26^,^27^ recommending outpatient management for patients with AUD, only two RCTs complied with these conditions, one being a two-armed RCT and the other involving only the group without antibiotic therapy. This is intriguing considering the extensive literature published in favor of outpatient regimes. Therefore, one of the most important primary outcomes in this setting, the need for readmission due to treatment failure, is obviously biased. However, it has been replaced with other variables like the need of readmission or progression of the degree of diverticulitis.

As an example, to palliate possible bias when analyzing readmission rate, the study by Mora López *et al*.^13^ considered the patients with repeated visits to the emergency department as readmissions despite them not being admitted to the hospital, while the rest of the studies consider only the patients visiting the emergency department or those admitted again after discharge as readmissions.

Despite the analyzed studies presenting great variability regarding the methodology used, all of them have in common the careful selection of patients and some strict criteria for the patients to be included in the clinical trials. In the four RCTs, immunocompromised patients, elderly patients, and those who present severity in terms of clinical signs or data from laboratory tests are excluded. Therefore, the sample for this study can be considered homogenous.

It is important to highlight the low rates of hospital readmission (5.3 and 7.1%) obtained for both groups, which is consistent with outpatient management generally being recommended in cases of AUD. In addition to this, the rates of patients requiring a change of therapeutic strategy are very low (4.1% in both groups) despite the criteria to define it not being sufficiently homogenous among the studies.

These results, together with the low percentage of patients that required emergency surgery or experienced a worsening in their radiological staging of the disease, endorse nonantibiotic regimes as effective and reliable treatments in selected AUD patients. Therefore, as supported by the most recent treatment guidelines for acute diverticulitis, therapeutic protocol without antibiotics should be the first option in cases of AUD patients that meet the established criteria.

In summary, the results obtained from this meta-analysis support previous findings and add new relevant evidence that endorses the use of therapeutic protocol without antibiotics in cases of AUD since the results from the Mora-López *et al.*’s^13^ study are included, which were not previously evaluated.

The limitations of this meta-analysis include the large variability in the methodologies used, which is reflected in the different antibiotherapy regimens, hospital admission criteria, or follow-up time, which can potentially alter the results of the analysis.

The existing evidence cannot be regarded as irrefutable, and therefore, the need for further research in this area is still justified^28^. It is hence necessary to globally engage in discussions regarding the means guide to try to establish homogeneous criteria in terms of outpatient management and what is the best regime of treatment without antibiotherapy.

Considering the results of this meta-analysis, further research is warranted to clarify some unsolved questions such as the best alternative treatment to antibiotics in strictly selected patients for these regimes, potential benefits of the antibiotics-free scheme in those groups of patients that did not fulfill inclusion criteria in previous trials and even the benefit in patients with chronic diverticulitis.

## Conclusions

This study shows that, in a highly selected subgroup of patients with AUD, nonantibiotic treatment is effective and safe. When analyzing the following outcome variables, readmission rates, need for emergency surgery, change of treatment strategy, clinical or radiological worsening or persistence of acute diverticulitis, comparing between conventional treatment regimens and nonantibiotic ones, no statistically significant differences were found. To the best of our knowledge, this is the only meta-analysis that encompasses the largest number of RCTs published to date and provides compelling evidence to justify further progress in this field. Further well-designed multicentre RCTs, incorporating more current antibiotic combinations for the management of these patients in the control group, more precisely defined treatment strategies in the experimental group, as well as incorporating outpatient management, are still needed to irrefutably confirm the efficacy and safety of nonantibiotic treatment for these selected groups of AUD patients. Research in other patients´ groups not included in these studies is still pending.

## Ethical approval

Ethical approval was not needed because this study is a meta-analysis and systematic review.

## Sources of funding

No sources of funding for our research were needed.

## Author contribution

A.C.B. and C.C.S.: conceptualization, data curation, formal analysis, investigation, methodology, project administration, validation, visualization, writing – original draft, writing – review and editing. M.D.M.: conceptualization, data curation, formal analysis, methodology, project administration, validation, visualization, writing – original draft, writing – review and editing. L.B.T. and A.G.Q.: writing – original draft, writing – review and editing. E.M.P.: supervision, validation, visualization. S.B.: supervision, validation, visualization, writing – original draft, writing – review and editing. J.G.S.: conceptualization, methodology, project administration, supervision, validation, visualization, writing – review and editing.

## Conflicts of interest disclosure

The authors declare that they have no financial conflict of interest with regard to the content of this report.

## Research registration unique identifying number (UIN)

CRD42021291317.

## Guarantors

Alba Correa Bonito and Carlos Cerdán Santacruz.

## Provenance and peer review

Not commissioned, externally peer-reviewed.

## References

[1] Weizman AV Nguyen GC . Diverticular disease: epidemiology and management. Can J Gastroenterol 2011;25:385–389. 21876861 10.1155/2011/795241 PMC3174080

[2] Parks TG . Natural history of diverticular disease of the colon. Clin Gastroenterol 1975;4:53–69. 1109820

[3] Beckham H Whitlow CB . The medical and nonoperative treatment of diverticulitis. Clin Colon Rectal Surg 2009;22:156–160. 20676258 10.1055/s-0029-1236159 PMC2780265

[4] Shahedi K Fuller G Bolus R . Long-term risk of acute diverticulitis among patients with incidental diverticulosis found during colonoscopy. Clin Gastroenterol Hepatol 2013;11:1609–1613. 23856358 10.1016/j.cgh.2013.06.020 PMC5731451

[5] Friend K Mills AM . Is outpatient oral antibiotic therapy safe and effective for the treatment of acute uncomplicated diverticulitis? Ann Emerg Med 2011;57:600–602. 21770056 10.1016/j.annemergmed.2010.11.008

[6] Tursi A Brandimarte G Giorgetti G . The clinical picture of uncomplicated versus complicated diverticulitis of the colon. Dig Dis Sci 2008;53:2474–2479. 18231855 10.1007/s10620-007-0161-2

[7] Hjern F Josephson T Altman D . Conservative treatment of acute colonic diverticulitis: are antibiotics always mandatory? Scand J Gastroenterol 2007;42:41–47. 17190761 10.1080/00365520600780650

[8] van Dijk ST Daniels L Ünlü Ç . Dutch Diverticular Disease (3D) Collaborative Study Group. Long-term effects of omitting antibiotics in uncomplicated acute diverticulitis. Am J Gastroenterol 2018;113:1045–1052. 29700480 10.1038/s41395-018-0030-y

[9] Isacson D Smedh K Nikberg M . Long-term follow-up of the AVOD randomized trial of antibiotic avoidance in uncomplicated diverticulitis. Br J Surg 2019;106:1542–1548. 31386199 10.1002/bjs.11239

[10] Chabok A Påhlman L Hjern F . AVOD Study Group. Randomized clinical trial of antibiotics in acute uncomplicated diverticulitis. Br J Surg 2012;99:532–539. 22290281 10.1002/bjs.8688

[11] Daniels L Ünlü Ç de Korte N . Dutch Diverticular Disease (3D) Collaborative Study Group. Randomized clinical trial of observational versus antibiotic treatment for a first episode of CT-proven uncomplicated acute diverticulitis. Br J Surg 2017;104:52–61. 27686365 10.1002/bjs.10309

[12] Jaung R Nisbet S Gosselink MP . Antibiotics do not reduce length of hospital stay for uncomplicated diverticulitis in a pragmatic double-blind randomized trial. Clin Gastroenterol Hepatol 2021;19:503–510.e1. 32240832 10.1016/j.cgh.2020.03.049

[13] Mora-López L Ruiz-Edo N Estrada-Ferrer O . DINAMO-Study Group. Efficacy and safety of nonantibiotic outpatient treatment in mild acute diverticulitis (DINAMO-study): a multicentre, randomised, open-label, noninferiority trial. Ann Surg 2021;274:e435–e442. 34183510 10.1097/SLA.0000000000005031

[14] Sartelli M Weber D Kluger Y . 2020 update of the WSES guidelines for the management of acute colonic diverticulitis in the emergency setting. World J Emerg Surg 2020;15:32. 32381121 10.1186/s13017-020-00313-4 PMC7206757

[15] Francis NK Sylla P Abou-Khalil M . EAES and SAGES 2018 consensus conference on acute diverticulitis management: evidence-based recommendations for clinical practice. Surg Endosc 2019:2726–2741. 31250244 10.1007/s00464-019-06882-z PMC6684540

[16] Schultz JK Azhar N Binda GA . European Society of Coloproctology: guidelines for the management of diverticular disease of the colon. Colorectal Dis 2020;22(suppl 2):5–28. 10.1111/codi.15140 32638537

[17] Page MJ McKenzie JE Bossuyt PM . The PRISMA 2020 statement: an updated guideline for reporting systematic reviews. Int J Surg 2021;88:105906. 33789826 10.1016/j.ijsu.2021.105906

[18] Shea BJ Bouter LM Peterson J . External validation of a measurement tool to assess systematic reviews (AMSTAR). PLoS One 2007;2:e1350. 18159233 10.1371/journal.pone.0001350 PMC2131785

[19] Levels of Evidence Working Group. “The Oxford 2011 Levels of Evidence”; 2011: Oxford Centre for Evidence-Based Medicine. Accessed 15 January 2022. http://www.cebm.net/index.aspx?o=5653

[20] Au S Aly EH . Treatment of uncomplicated acute diverticulitis without antibiotics: a systematic review and meta-analysis. Dis Colon Rectum 2019;62:1533–1547. 30663999 10.1097/DCR.0000000000001330

[21] Desai M Fathallah J Nutalapati V . Antibiotics versus no antibiotics for acute uncomplicated diverticulitis: a systematic review and meta-analysis. Dis Colon Rectum 2019;62:1005–1012. 30664553 10.1097/DCR.0000000000001324

[22] Garfinkle R Salama E Amar-Zifkin A . Observational versus antibiotic therapy for acute uncomplicated diverticulitis: a non-inferiority meta-analysis based on a Delphi consensus. Surgery 2022;171:328–335. 34344525 10.1016/j.surg.2021.07.012

[23] Brochmann ND Schultz JK Jakobsen GS . Management of acute uncomplicated diverticulitis without antibiotics: a single-centre cohort study. Colorectal Dis 2016;18:1101–1107. 27089051 10.1111/codi.13355

[24] Estrada Ferrer O Ruiz Edo N Hidalgo Grau LA . Selective non-antibiotic treatment in sigmoid diverticulitis: is it time to change the traditional approach? Tech Coloproctol 2016;20:309–315. 27053254 10.1007/s10151-016-1464-0

[25] Sartelli M Chichom-Mefire A Labricciosa FM . The management of intra-abdominal infections from a global perspective: 2017 WSES guidelines for management of intra-abdominal infections. World J Emerg Surg 2017;12:29. 28702076 10.1186/s13017-017-0141-6 PMC5504840

[26] Cirocchi R Randolph JJ Binda GA . Is the outpatient management of acute diverticulitis safe and effective? A systematic review and meta-analysis. Tech Coloproctol 2019;23:87–100. 30684110 10.1007/s10151-018-1919-6

[27] Biondo S Golda T Kreisler E . Outpatient versus hospitalization management for uncomplicated diverticulitis: a prospective, multicenter randomized clinical trial (DIVER Trial). Ann Surg 2014;259:38–44. 23732265 10.1097/SLA.0b013e3182965a11

[28] Boutros M . Outpatient treatment of uncomplicated diverticulitis with either antibiotic or nonantibiotic treatment (MUD). Identifier: NCT03146091. Accessed 4 January 2018. https://clinicaltrials.gov/ct2/show/

